# Do GnRH Agonists Really Increase Body Weight Gain? Evaluation of a Multicentric Portuguese Cohort of Patients With Central Precocious Puberty

**DOI:** 10.3389/fped.2022.816635

**Published:** 2022-03-04

**Authors:** Ana Luísa Leite, Elisa Galo, Ana Antunes, Brígida Robalo, Daniela Amaral, Filipa Espada, Sofia Castro, Sara Simões Dias, Catarina Limbert

**Affiliations:** ^1^Unidade de Endocrinologia e Diabetologia Pediátrica, Centro Hospitalar de Vila Nova de Gaia/Espinho, EPE, Espinho, Portugal; ^2^Serviço de Pediatria, Departamento de Pediatria, Hospital da criança e do Adolescente, Hospital da Luz, Lisboa, Portugal; ^3^Unidade de Endocrinologia Pediátrica, Hospital de Braga, Braga, Portugal; ^4^Unidade de Endocrinologia, Serviço de Pediatria, Departamento de Pediatria, Hospital de Santa Maria, Centro Hospitalar Universitário Lisboa Norte, Lisboa, Portugal; ^5^Clínica Universitária de Pediatria, Faculdade de Medicina, Universidade de Lisboa, Lisboa, Portugal; ^6^Serviço de Pediatria, Hospital Lusíadas Lisboa, Lisboa, Portugal; ^7^Unidade de Endocrinologia Pediátrica, Hospital Pedro Hispano, Matosinhos, Portugal; ^8^Serviço de Pediatria, Centro Hospitalar Barreiro Montijo, Barreiro, Portugal; ^9^EpiDoc Nova Medical School, Faculdade Ciências Médicas, Universidade Nova de Lisboa, Lisboa, Portugal; ^10^ciTechCare, Center for Innovative Care and Health Technology, Escola Superior de Saúde de Leiria, Politécnico de Leiria, Leiria, Portugal; ^11^Unidade de Endocrinologia Pediátrica, Centro Hospitalar Universitário de Lisboa Central, Lisboa, Portugal; ^12^Nova Medical School, Universidade Nova de Lisboa, Lisboa, Portugal

**Keywords:** central precocious puberty (CPP), GnRH agonists, weight gain (WG), body mass index (BMI), obesity

## Abstract

**Introduction:**

There are several concerns associated with gonadotropin-releasing hormone agonist (GnRHa) treatment for central precocious puberty (CPP), such as obesity and changes in body mass index (BMI). We aimed to investigate whether any anthropometric differences exist and if they persist over time.

**Methods:**

We conducted an observational study of Portuguese children (both sexes) diagnosed with CPP between January 2000 and December 2017, using a digital platform, in order to analyze the influence of GnRHa treatment on BMI-SD score (BMI-SDS).

**Results:**

Of the 241 patients diagnosed with CPP, we assessed 92 patients (8% boys) in this study. At baseline, 39% of the patients were overweight. BMI-SDS increased with treatment for girls but then diminished 1 year after stopping GnRHa therapy (*p* = 0.018). BMI-SDS variation at the end of treatment was negatively correlated with BMI-SDS at baseline (*p* < 0.001). Boys grew taller and faster during treatment than did girls (*p* < 0.001), and therefore, their BMI-SDS trajectory might be different.

**Conclusions:**

This study showed an increase of body weight gain during GnRHa treatment only in girls, which reversed just 1 year after stopping treatment. The overall gain in BMI-SDS with treatment is associated with baseline BMI-SDS.

## Introduction

Central precocious puberty (CPP) results from the premature activation of the hypothalamic–pituitary–gonadal axis. It mimics physiological pubertal development but occurs at an inappropriate chronological age (1). It is defined by the onset of pubertal development before the age of 8 and 9 years in girls and boys, respectively (2).

In recent decades, there have been global reports of a secular trend toward the earlier onset of puberty in the general population, but disconcertingly, it seems that CPP is also increasing (3).

Early recognition of CPP facilitates appropriate and prompt intervention such as treatment with gonadotropin-releasing hormone agonists (GnRHas) or surgical intervention in certain cases such as those involving central nervous system tumors (4, 5). GnRHas are synthetic peptide analogs of hypothalamic GnRH used to suppress puberty and have been the standard of care for more than two decades for children with CPP (6). GnRHa preparations help preserve adult height (AH) and prevent/ameliorate the presumed distress associated with early maturation and menarche in girls (7).

However, the possible effects of body weight gain, obesity, and untimely metabolic changes are the major concerns associated with GnRHa use (8–10). The available data are inconsistent owing to the heterogeneity of study protocols, the small number of patients, and the exclusion of boys from previous studies (11).

Some studies have reported an association between GnRHa treatment and body mass index (BMI) increase (9, 11–13), in previously normal-weight girls (14, 15) as well as in those with obesity (16). Other studies have found no influence of GnRHa treatment on weight status (8, 17, 18), and recently, it has been proposed that the increase of BMI occurs during treatment but reverts to baseline after GnRHa is discontinued (19, 20). In the literature, only one study was conducted in boys and reported no significant change in BMI-SDS over 2 years of therapy (21).

We aimed to evaluate sex-based differences and changes in BMI in a nationally representative group of Portuguese CPP patients treated with GnRHa and followed up for a year after treatment discontinuation.

## Materials and Methods

### Study Design

This was an observational multicentric study of children diagnosed with CPP in Portugal between January 2000 and December 2017. A digital platform was designed to record data, and eleven pediatric endocrinology centers throughout Portugal were enrolled.

The study was approved by the Portuguese Ethics Committee (CNPD no. 1704). The research was performed in adherence to the principles laid out in the *Declaration of Helsinki*.

The diagnosis of CPP was clinically defined as Tanner stage II breast development in girls younger than 8 years or testicular enlargement 4 ml or greater in boys younger than 9 years with baseline luteinizing hormone (LH) level ≥0.3 IU/L or GnRHa-stimulated LH peak >5 IU/L (22). Idiopathic and other cases of CPP were included.

The exclusion criteria were as follows: (1 not receiving treatment with GnRH agonists or not having yet completed treatment; (2 use of known weight-modifying medications (e.g., antipsychotics, stimulants, and steroids); and (3 having a weight-modifying illness (e.g., inflammatory bowel disease and type 1 diabetes) or unavailability of relevant data (including no baseline anthropometric data or no follow-up data).

From an initial cohort of 241 children diagnosed with CPP, a total of 92 patients were analyzed.

Anthropometric data were collected at baseline (*Time I*), at the end of GnRHa treatment (*Time II*), and 1 year after ending treatment with GnRHa formulations (*Time III*).

Baseline data included age at the onset of the first symptoms, clinical manifestation, age at the time of treatment initiation, sex, mid-parental height, Tanner stage, bone age (BA), GnRHa formulation and related medical information, previous relevant medical records, cause of CPP, and anthropometric measures. Body weight was measured in kilograms using a digital weighing scale with a sensitivity of 0.1 kg, and the height was measured in meters with a stadiometer, in the standing position, with a 0.1-cm sensitivity, both evaluated by trained medical staff. BMI was calculated as weight (in kilograms) divided by height (in meters) squared (absolute value). BMI-SD score [SDS] was calculated using *Anthro* software (WHO, Geneva, Switzerland; and CDC, USA) (23).

To allow a better understanding of anthropometric changes, we evaluated BMI categories according to the WHO classification: normal weight, between the 10th and 85th percentiles; overweight, between the 85th and 95th percentiles; and obesity, 95th percentile or greater. We had no children at <5th percentile BMI in our study.

We calculated the change in BMI-SDS from baseline to the end of treatment (ΔTime II–I) and from baseline to a year after ending treatment (ΔTime III–I).

To evaluate height changes over the course of treatment, we also calculated the change in height-SDS at Time II and at Time I compared with baseline.

### Statistical Analysis

Statistical analysis was performed using the SPSS™ version 26.0 software (SPSS Inc., Chicago, IL, USA). All probabilities were two-tailed, and *p*-values < 0.05 were regarded as significant. Quantitative variables were described as mean ± SD and qualitative variables as absolute frequencies and percentages. The Kolmogorov–Smirnov test was performed to assess whether data were distributed normally. Student's *t-*test and ANOVA were used when variables were normally distributed; and when data were not normally distributed, we used the Mann–Whitney test. Qualitative variables were compared using the chi-square or Fisher's exact test. Linear regression models were used to study the association between BMI-SDS at the end of treatment (Time II) and BMI-SDS at baseline (Time I) after adjusting for age, sex, and duration of treatment.

## Results

A total of 241 children were diagnosed with CPP during the time of the study, but only 92 were eligible for study participation. We report data after 1 year of completing GnRHa treatment (Time III) in 64 cases. Of the 92 patients enrolled, the majority were girls (*n* = 84; 91%). Of the eight boys included (8%), all had completed follow-up until 1 year after stopping treatment. Baseline characteristics of study participants are summarized in Table 1.

**Table 1:** *Time I* (T1 = baseline) clinical characteristics of the patients and gender comparison.

	**Total** ***n =* 92**	**Gender**		***p***
		**Girls** ***n =* 84**	**Boys** ***n =* 8**	
Age of symptoms (y)	6.5 ± 1.6	6.4 ± 1.6	7.4 ± 1.8	N.S.
Clinical manifestation^*^				
Thelarche/Increase Testicular Volume	35 (38%)	34 (39%)	1 (11%)	
Menarche	3 (2%)	3 (3%)	-	N.S.
Pubarche	32 (34%)	28 (35%)	3 (67%)	
Increase in Growth Velocity	22 (24%)	19 (12%)	4 (22%)	
Tanner stage at baseline^*^				
Tanner II	54 (62%)	47 (60%)	7 (87%)	
Tanner III	21 (25%)	20 (26%)	1 (13%)	
Tanner IV	11 (12%)	10 (13%)	-	**0.034**
Tanner V	1 (1%)	1 (1%)	-	
Age of starting treatment (y)	7.8 ± 1.6	7.7 ± 1.6	8.6 ± 1.5	N.S.
Time I weight (kg)	33.8 ± 8.4	33.1 ± 7.6	40.9 ± 12.8	N.S.
Time I weight-SDS	1.4 ± 1.2	1.3 ± 1.0	2.0 ± 2.4	N.S.
Time I stature (cm)	134.2 ± 11.6	134.0 ± 11.3	136.1 ± 15.3	N.S.
Time I stature-SDS	1.2 ± 1.3	1.3 ± 1.2	0.8 ± 2.4	N.S.
Time I BMI (Kg/m2)	18.5 ± 2.9	18.2 ± 2.6	21.2 ± 4.2	**0.002**
Time I BMI-SDS	1.1 ± 1.2	0.9 ± 1.1	2.2 ± 1.6	**0.005**
Time I growth velocity (cm/y)	8.2 ± 2.2	8.3 ± 2.3	7.7 ± 1.8	N.S.
Target height-SDS	−0.3 ± 0.9	−0.32 ± 0.9	−0.41 ± 1.1	N.S.

* *Chi-square test. Bold values are p < 0.05*.

Their mean age at onset of symptoms was 6.5 ± 1.6 years, and the majority presented with thelarche/gonadarche. At baseline, girls showed a more advanced Tanner stage (*p* = 0.034) and initiated GnRHa treatment about 1.3 years after the onset of puberty signs. Boys were heavier than girls before treatment (girls BMI, 18.2 ± 2.6, and boys BMI, 21.2 ± 4.2, *p* = 0.002; and girls BMI-SDS, 0.9 ± 1.1, and boys BMI-SDS, 2.2 ± 1.6, *p* = 0.005). Our sample had a total of 81 white patients and 11 (12%) non-white patients. The 11% non-white participants were all girls. Idiopathic CPP was the main diagnosis (90%), with only 9 cases of secondary CPP (3 boys; 33% of secondary cases).

At baseline, less than half of the participants had normal BMI (42; 46%), 36 were overweight (39%), and 14 had obesity (15%), with differences noticed between sexes (4 boys, of whom 2 had obesity; *p* = 0.035).

**Figure 1:**
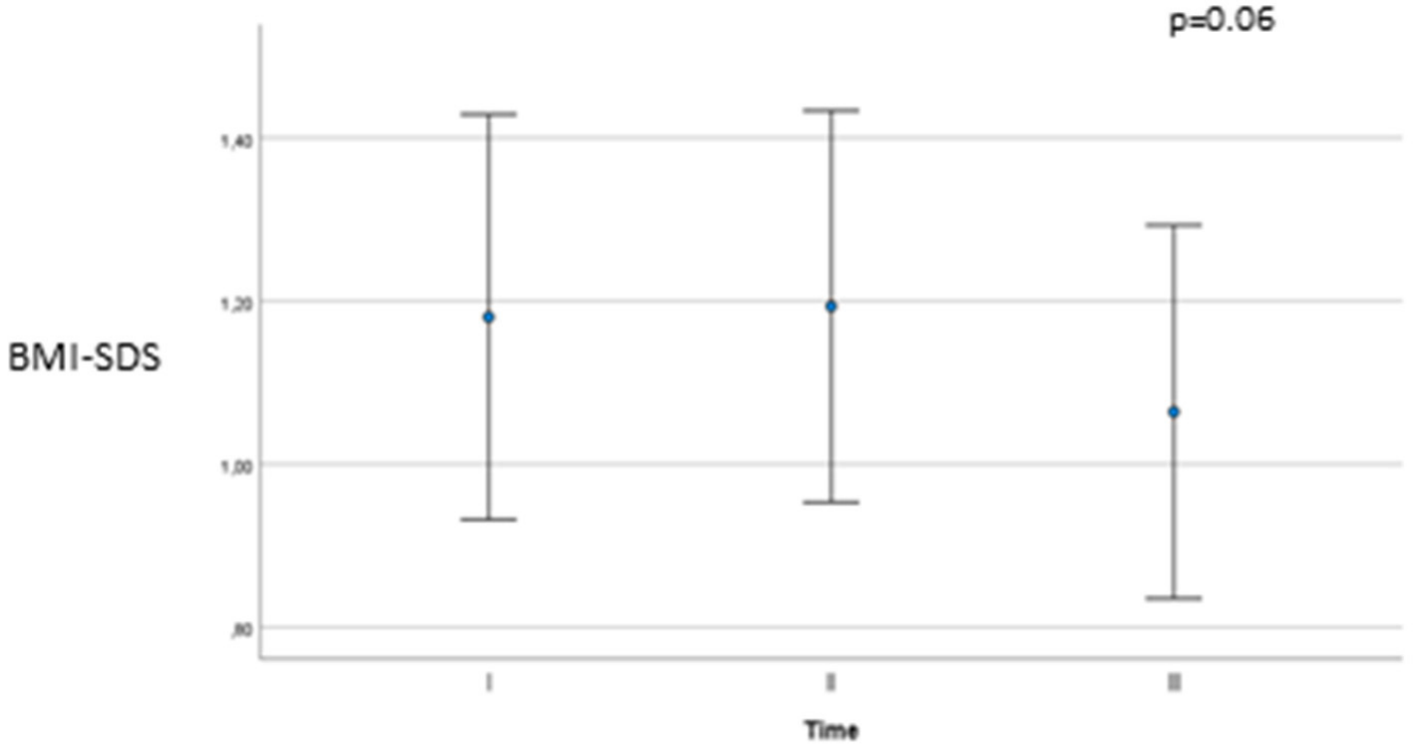
BMI-SDS at baseline (Time I), end of treatment (Time II) and one year after stopping GnRH agonists (Time III).

BMI-SDS tended to increase with treatment but decreased a year after stopping GnRHa therapy (Figure 1: BMI-SDS 1.18 ± 1.0 at time I; 1.19 ± 1.0 at time II; and 1.06 ± 0.9 at time III; *p* = 0.06). To better understand this evolution, we analyzed changes in BMI-SDS over study duration (Table 2). BMI-SDS increased (0.013 ± 0.67) from baseline to time I and decreased (−0.12 ± 0.66; p = 0.032) from baseline to time III.

**Table 2:** Variations in BMI-SDS along study time according to sex.

	**BMI-SDS Δ Time II-I** ** (*n =* 64)**		**BMI-SDS Δ Time III-I** ** (*n =* 64)**	***p***
Total	0.013 ± 0.67		−0.12 ± 0.66	0.032
	**BMI-SDS** **Δ** **Time II-I**	***p***	**BMI-SDS** **Δ** **Time III-I**	***p***
Girls	0.13 ± 0.63	0.027	−0.04 ± 0.68	0.018
Boys	−0,40 ± 0.72		−0.63 ± 0.54	

Girls and boys differed such that the increase in BMI-SDS was noted in girls, but not boys (*p* = 0.027). Both sexes had a decrease in BMI-SDS from baseline to time III, but this decrease was greater in the boys (*p* = 0.012). Thus, BMI-SDS increased during treatment and decreased a year after the end of treatment in girls. In contrast, BMI-SDS decreased both during treatment and a year after the end of GnRHa treatment in boys.

A multivariable linear regression was computed to evaluate the independent predictors of change in BMI SDS from baseline to time II–I. After adjustment for age, sex, and duration of GnRHa use, BMI-SDS at baseline was associated negatively with the change from baseline to time II in those who were overweight or had obesity (β = −0.386, *p* = 0.005).

**Figure 2:**
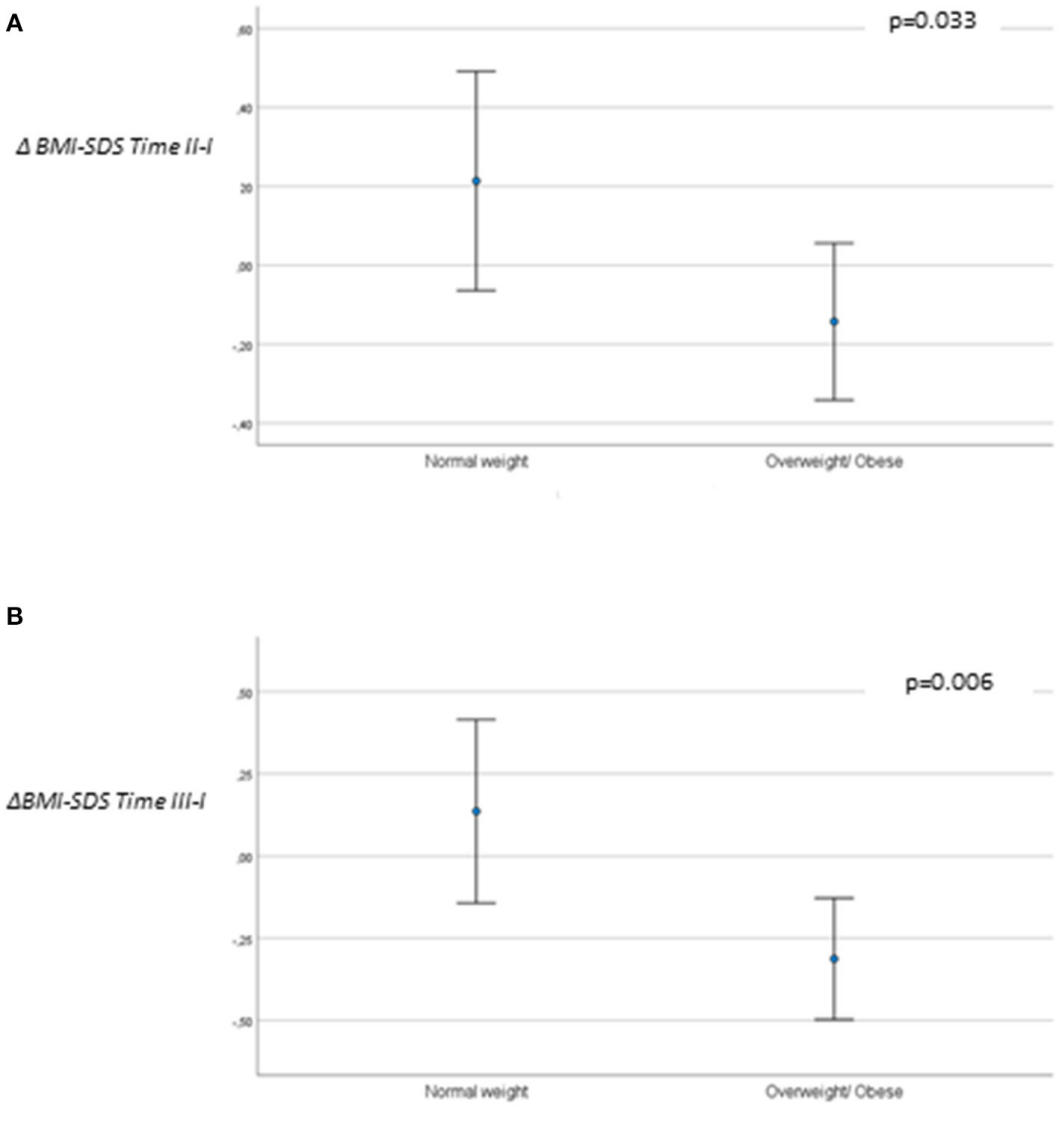
**(A)** BMI-SDS variation Δ*Time II–I* and **(B)** BMI-SDS variation Δ*Time III–I*, according to BMI group at baseline (*n* = 64).

Considering BMI categories at baseline and progression over time, BMI-SDS significantly increased in the normal weight group compared with the group that was overweight or had obesity (ΔTime II–I, p = 0.033; and ΔTime III–I, *p* = 0.006) (Figures 2A,B).

**Figure 3:**
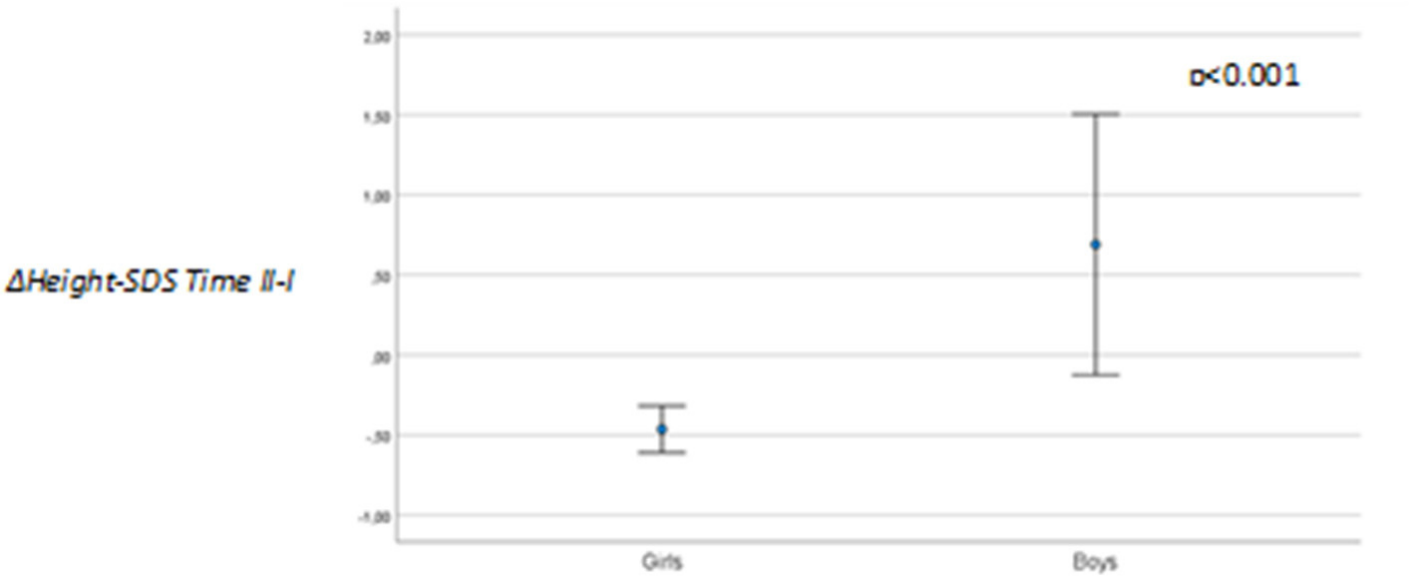
Height-SDS variation (Δ*Time II–I*) between genders (*n* = 92).

Focusing on the effect of the agonists on growth, we observed a statistically significant difference in the variation of height-SDS during treatment between boys and girls (girls, −0.48 ± 0.67; boys, 0.69 ± 0.97; *p* < 0.001) (Figure 3).

## Discussion

In this study, we observed an increase in BMI-SDS during treatment with GnRH agonists in girls, but not in boys. However, this increase was temporary and reversed just 1 year after stopping treatment for the group as a whole. The overall treatment-related increase in BMI-SDS was inversely related to the pretreatment BMI. Therefore, the higher the BMI-SDS before the treatment, the less the weight gain is.

Few studies describe what happens when boys with CPP receive treatment. In our study, we found that the trend for gain in BMI-SDS is similar for boys and girls. The main difference is in height increment during treatment, which seems better controlled in girls. In the last 20 years, due to the increase in the prevalence of GnRHa use, several studies have started analyzing their impact not only on AH but also on BMI changes.

Yoon et al. described that in girls, overall, BMI-SDS for chronological age did not change significantly during or after GnRHa treatment discontinuation, regardless of their pretreatment BMI (22). These results contradict those reported by the Spanish PUBERE Group who noted that during treatment with GnRHa, girls experience a significant increase in BMI-SDS that persists after therapy is discontinued and AH has been reached (11). There are several conflicting findings, which are possibly attributable to the differences in race or ethnicity. In addition, differences in study protocols may also contribute to the conflicting data. In 2020, Vuralli et al. showed that BMI-SDS increased during GnRHa treatment only in girls in the “normal weight” category and not in those considered “overweight” and that this effect was reversible following treatment discontinuation (19).

In our study, we noted a similar increase in BMI, but we also observed its resolution and reversal after only 1 year of termination of the GnRHa therapy. As reported previously, our data demonstrated that the pattern of change in BMI-SDS varies according to the individual's BMI (14, 17). One explanation might be that fatty mass redistribution/deposition due to gonadal axis suppression is more significant in lean children than in overweight or obese children. The pharmacological interruption of the LH/follicle-stimulating hormone (LH/FSH) axis with suppression of estrogen production may be responsible for the enhanced deposition of adipose tissue in lean children compared with overweight or obese ones (23). The evidence of a transient effect on weight gain due to gonadal axis interruption leads us to propose that all patients with CPP on GnRHa therapy and their caregivers should be informed about weight management and that recommendations for lifestyle changes should be reinforced at every appointment, independent of BMI category (although effects are certainly most marked in those with lower BMI-SDS at treatment onset).

Our study is one of the very few studies that have enrolled boys treated with GnRHa for CPP and to our knowledge the only one that analyses what happens to these boys 1 year after stopping treatment. We demonstrate that the change in height-SDS significantly differs with sex, similar to the results found by Lee et al. (6). One explanation for this difference might be the pubertal growth spurt, which occurs at the beginning of puberty in girls and in the middle of puberty in boys (25). In addition, our results clearly show different BMI-SDS responses in girls and boys. This is probably due to a greater interruption of height increase in girls, which also contributes to the increase in BMI, given that BMI is calculated as weight (kg) divided by height (meters) squared. Leptin levels are positively related to adiposity and negatively related to testosterone levels. Additionally, blood leptin levels differ across the sexes through puberty to reach higher levels in girls than in boys (24–26). This would explain why BMI does not increase in boys as much as it does in girls during CPP treatment.

A limitation of this study is its retrospective nature, and consequently, we could not ensure that all patients were given similar dietary recommendations. Further, the number of boys in the study was small, and a larger number of boys would be necessary to further validate the different responses we noted between sexes to GnRHa treatment.

The multicentric CPP cohort with national representation and follow-up at 1 year after treatment in both boys and girls is the strength of our study.

## Conclusions

The treatment of CPP with GnRHa causes a transient increase in BMI-SDS with a return to the basal BMI-SDS a year after stopping GnRHa. The increase in BMI-SDS is more evident in children with normal weight before treatment, but this effect is reversible and does not induce obesity.

Boys respond differently (with a lower BMI increase) to GnRH agonists.

National databases of CPP individuals are an important tool for conducting long-term follow-up studies and assessing anthropometric and metabolic variables that may be affected by the interruption of the hypothalamic–pituitary–gonadal axis.

Families and healthcare providers should be made aware of the importance of nutritional status and healthy lifestyles in CPP children.

## Data Availability Statement

The raw data supporting the conclusions of this article will be made available by the authors, without undue reservation.

## Ethics Statement

The studies involving human participants were reviewed and approved by Portuguese Ethics Committee (CNPD n° 1704/2015). Written informed consent to participate in this study was provided by the participants' legal guardian/next of kin.

## Author Contributions

AL and EG wrote the first draft of the manuscript. SS, AL, and EG performed the analysis. SS draw the charts. CL undertook extensive critical review of the manuscript, but all authors together conceptualized the idea and approved the submission of the manuscript.

## Conflict of Interest

The authors declare that the research was conducted in the absence of any commercial or financial relationships that could be construed as a potential conflict of interest.

## Publisher's Note

All claims expressed in this article are solely those of the authors and do not necessarily represent those of their affiliated organizations, or those of the publisher, the editors and the reviewers. Any product that may be evaluated in this article, or claim that may be made by its manufacturer, is not guaranteed or endorsed by the publisher.
